# Gender disparities in kidney replacement therapies and transplantation in Colombia

**DOI:** 10.1186/s12882-024-03492-3

**Published:** 2024-02-26

**Authors:** Laura Nino-Torres, Jessica Pinto-Ramirez, Fernando Giron-Luque, Alejandro Nino-Murcia

**Affiliations:** 1 Department of Transplantation Surgery, Colombiana de Trasplantes, Av Carrera, 30 No. 47 A-74, Bogotá, 111311 Colombia; 2 Department of Transplantation Nephrology, Colombiana de Trasplantes, Bogotá, Colombia

**Keywords:** Gender disparity, Organ transplantation, CKD, Diabetes, Renal replacement therapy

## Abstract

**Background:**

In chronic kidney disease (CKD), there are historical inequities in multiple stages of the pathway for organ transplantation. Women have been recognized as disadvantaged within this process even after several efforts. Therefore, we aimed to analyze the prevalence and incidence of CKD by gender and their access to Kidney replacement therapy (KRT) in Colombia.

**Methods:**

A cross-sectional study based on secondary analysis of national information on CKD, hypertension, diabetes, waiting list, deceased, and living donor transplantation between 2015 and 2020.

**Results:**

In Colombia, 4.934.914 patients were diagnosed with hypertension, diabetes, or CKD. 60,64% were female, with a mean age of 63.84 years (SD 14,36). Crude incidence for hypertension (10.85 vs. 7.21 /1000 inhabitants), diabetes mellitus (3.77 vs. 2.98 /1000 inhabitants), and CKD (4 vs. 2 /1000 inhabitants) was higher for females. Crude incidence for KRT was 86.45 cases /100.0000 inhabitants. In 2020, 2978 patients were on the waiting list, 44% female. There were 251 deaths on the waiting list, 38% female. This year, 517 kidney transplants were performed, and only 40% were female.

**Conclusion:**

In Colombia, there are proportionally more females with CKD and precursor comorbidities. Nevertheless, there are fewer females on the waiting list and transplanted annually.

**Supplementary Information:**

The online version contains supplementary material available at 10.1186/s12882-024-03492-3.

## Introduction

In chronic kidney disease (CKD) there are historical inequities in multiple stages of the pathway for organ transplantation [1]: access to waiting lists, organ distribution of deceased donor kidneys, living donor kidney transplant recipients, and preemptive transplantation [2–4]. Among several non-medical factors that have been described that can affect access to transplants, gender is one of them [5]. Since men are favored in the process, a gender inequity is created in which women have been recognized as disadvantaged even after several efforts [5]. This inequity has been repetitively proven through data analysis of the US databases [6, 7]. It has been a common interest worldwide to recognize the impact of gender on equity and access to transplantation. In Colombia, there is a gendered difference in access to waiting lists, kidney allocation of cadaveric donors, and living donor recipients; although this has never been analyzed from information published by a local entity in Colombia.

Since 2007, Colombia has established as mandatory a national report of CKD including ESKD called *Cuenta de Alto Costo*, a database which in turn publishes once a year the epidemiological data regarding these pathologies [8]. Nevertheless, a detailed analysis of the data presented, including a comparison with gender distribution in our population has not been made, as a starting point to find different ways to close the gap between men and women when regarding organ transplantation.

In this study, we aim to describe prevalence, incidence and mortality of CKD broken down by gender and the possibility of kidney replacement therapy (KRT) access including kidney transplantation (KT) in Colombia. Additionally, we aim to describe the prevalence, incidence and mortality of chronic arterial hypertension and diabetes as precursors of end stage kidney disease (ESKD) by gender, the number of patients involved in different modalities of KRT (peritoneal dialysis or hemodialysis), and mortality registered in this population. We also seek to detail the proportion of living donors and cadaveric donors by gender and KT patients. We will compare this by gender with Colombian population pyramids.

## Materials and methods

A cross-sectional study based on secondary analysis of local public information between 2015 and 2020 [8], which encompasses data pertaining to End-Stage Kidney Disease (ESKD), chronic arterial hypertension, and diabetes, encompassing statistics related to waiting lists, deceased and living kidney donor transplantation. The information is sourced from authoritative entities such as the *Departamento Administrativo Nacional de Estadística* (DANE), the National Health Institute, and the *Cuenta de Alto Costo* (CAC), an organization that systematically releases annual data concerning chronic diseases [9]. Patients older than 18 years were included. Categorical variables were analyzed through frequencies and percentages, while measures of central tendency (mean and/or median) and measures of dispersion (standard deviation and/or interquartile range, based on variable distribution) were computed. Microsoft Excel was employed to establish and analyze a dedicated database for recording information.

## Results

### Colombian population and demographics

Colombia is a country of South America which has a population of 50.882.884 inhabitants according to the census made on 2020, data registered by the *Departamento Administrativo Nacional de Estadística (DANE).* Gender wise, 50.9% are female and 49.1% are male (www.dane.gov.co). This difference is statistically significant (*p* = 0.016), while 68.2% are in the ages between 15 and 65 years old.

### Chronic arterial hypertension and diabetes mellitus

Chronic arterial hypertension and diabetes mellitus have been included in the diseases to report to the National health institute and the *Cuenta de alto costo*, an organization which annually publishes data regarding these amongst others. According to the 2020 report, 3.227,788 inhabitants have only chronic arterial hypertension, 331,520 inhabitants have only diabetes and 963,420 inhabitants have both coexisting diseases [10].

**Fig. 1:**
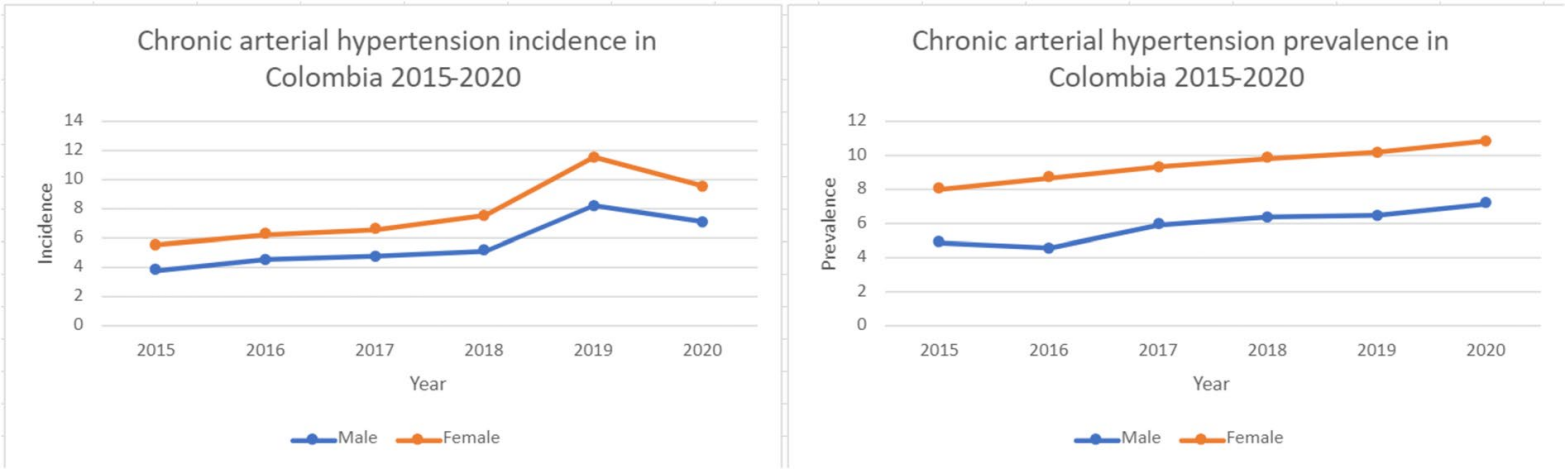
Chronic arterial hypertension incidence and prevalence in Colombia 2015–2020

When stratifying by gender, chronic arterial hypertension incidence is greater in females than males, and for 2019 and 2020 the difference is increasing with the new cases diagnosed each year. This is reflected in the prevalence of the disease, in which again females are more affected by the disease when compared to male patients (Fig. 1).

**Fig. 2:**
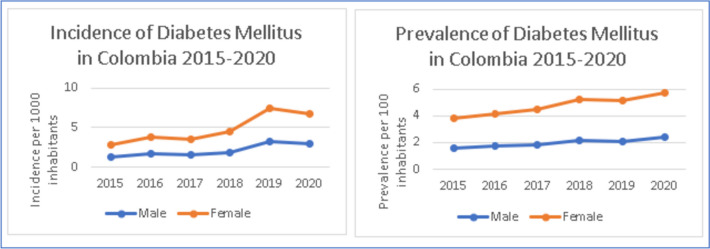
Incidence and prevalence of diabetes mellitus in Colombia 2015-2020

 Diabetes mellitus has shown a similar tendency when stratified by gender. Incidence is greater in females than males constantly through the years with 56.6% of new cases being female in 2020 (*n* = 105,597). Also, prevalence shows the same pattern throughout the years (Fig. 2).

### CKD in Colombia

The CKD population and especially ESKD has been growing over time [6]. To this day, CKD is considered a mayor health concern, affecting approximately 9.1% of the adult population worldwide [5, 11]. Nevertheless, prevalence of CKD has shown substantial differences between men and women, as well as the rate of progression [4, 6]. CKD prevalence worldwide has been reported to be higher in female, with the exception of Japan and Singapore [4, 7].

Colombia, by 2019 had registered 925,966 patients with chronic kidney disease, divided into the five stages of the disease [10]. Which represents a prevalence of 1.8% (Table 1).

**Table 1:** CKD patients by stage of the disease

Stages of CKD	Number of patients	% Of CKD patients	CKD Prevalence
Stage 1	151,262	16%	0.30%
Stage 2	199,319	22%	0.39%
Stage 3	471,715	51%	0.93%
Stage 4	58,085	6%	0.11%
Stage 5	45,615	5%	0.09%

**Fig. 3:**
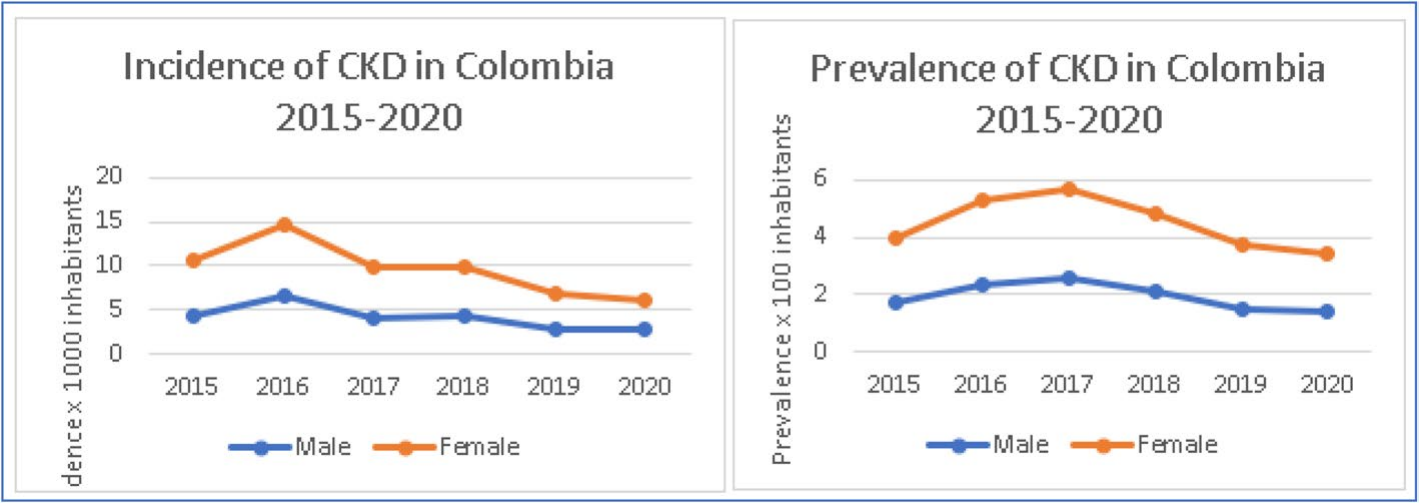
CKD incidence and prevalence in Colombia, 2015–2020

Stratifying by gender, CKD is both more incident and more prevalent in female patients (Fig. 3).

**Fig. 4:**
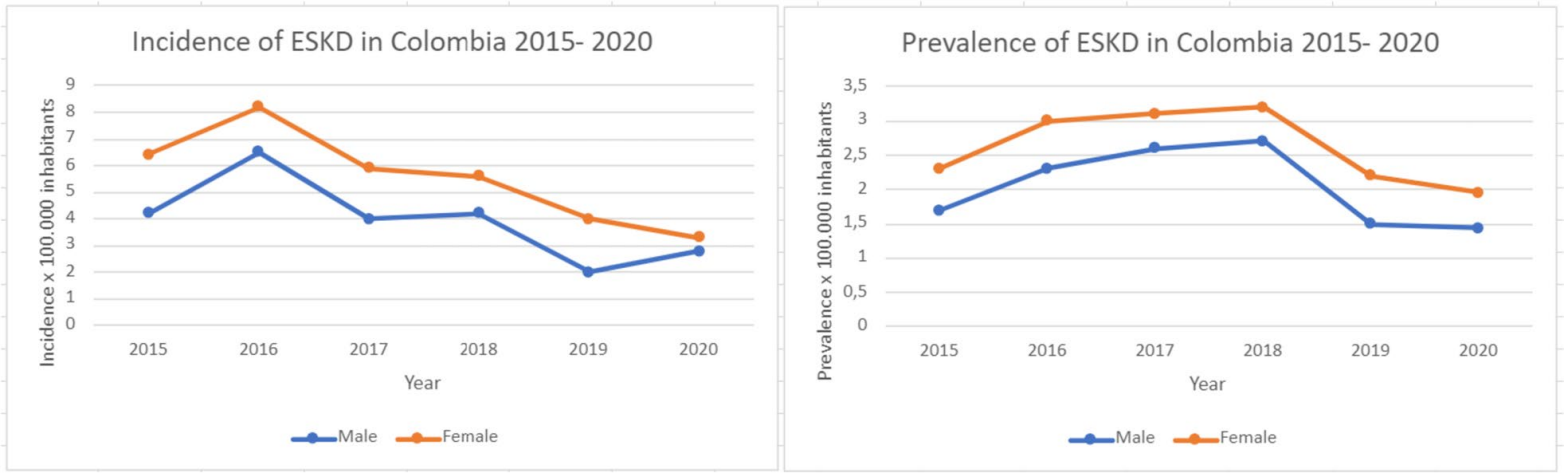
Incidence and prevalence of ESKD in Colombia, 2015–2020

Nevertheless, ESKD (Stage 5) has a different behavior when analyzed by gender. Incidence of ESKD is higher amongst female, as is prevalence between the years 2015–2020 (Fig. 4).

### Kidney replacement therapies in Colombia

In Colombia, there are 43,123 patients in KRT. Patients predominantly receive hemodialysis (*n* = 25,121), followed by peritoneal dialysis (*n* = 9,390). 761 patients with medical indication (Stage 5 CKD) have not started dialysis due to barriers in accessing healthcare. Therefore, only 7,734 patients have received KT.

**Fig. 5:**
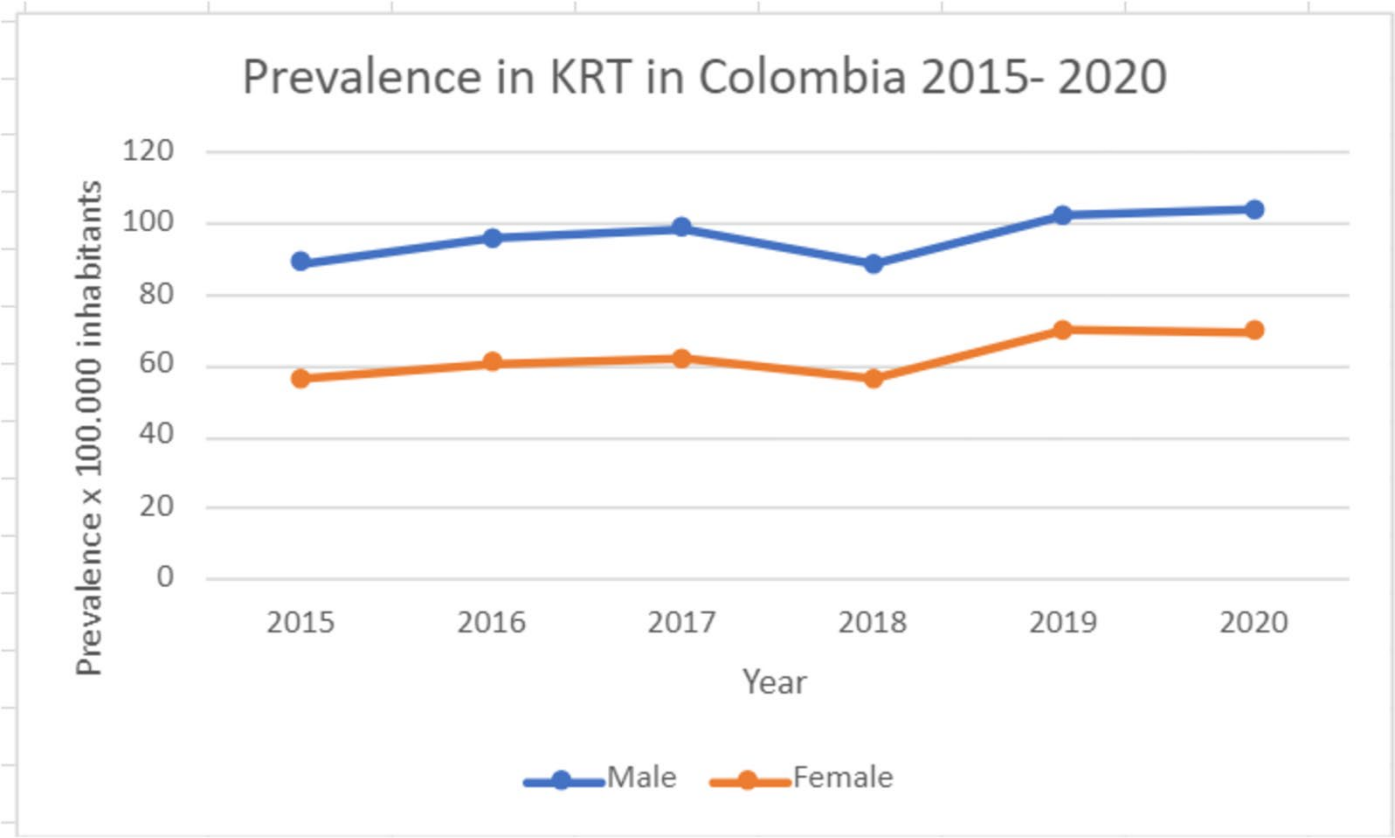
Prevalence of KRT in Colombia, 2015–2020

Access to KRT has been shown to be inequitable in different parts of the world, between women and men [6]. In Colombia, this has been reflected in the prevalence of KRT, in which there are proportionally more male than female patients (Fig. 5).

When analyzing the KRT modality in the Colombian population, either in peritoneal dialysis or hemodialysis, there is consistently a greater proportion of males when compared to female patients (Table 2).

**Table 2:** KRT by modality, number of patients, mortality and proportion of female patients

KRT modality	Number of patients	Women n (%)	Mortality in women n (%)
Peritoneal dialysis	9,390	4,357 (46,4%)	503 (46,7%)
Hemodialysis	25,121	9,740 (38,8%)	1288 (40,6%)

Mortality has also been recorded to be greater in males than in females in either modality of KRT in Colombia. This is also true for peritoneal dialysis patients with mortality of 46,7%.

### Access to waiting list

**Fig. 6:**
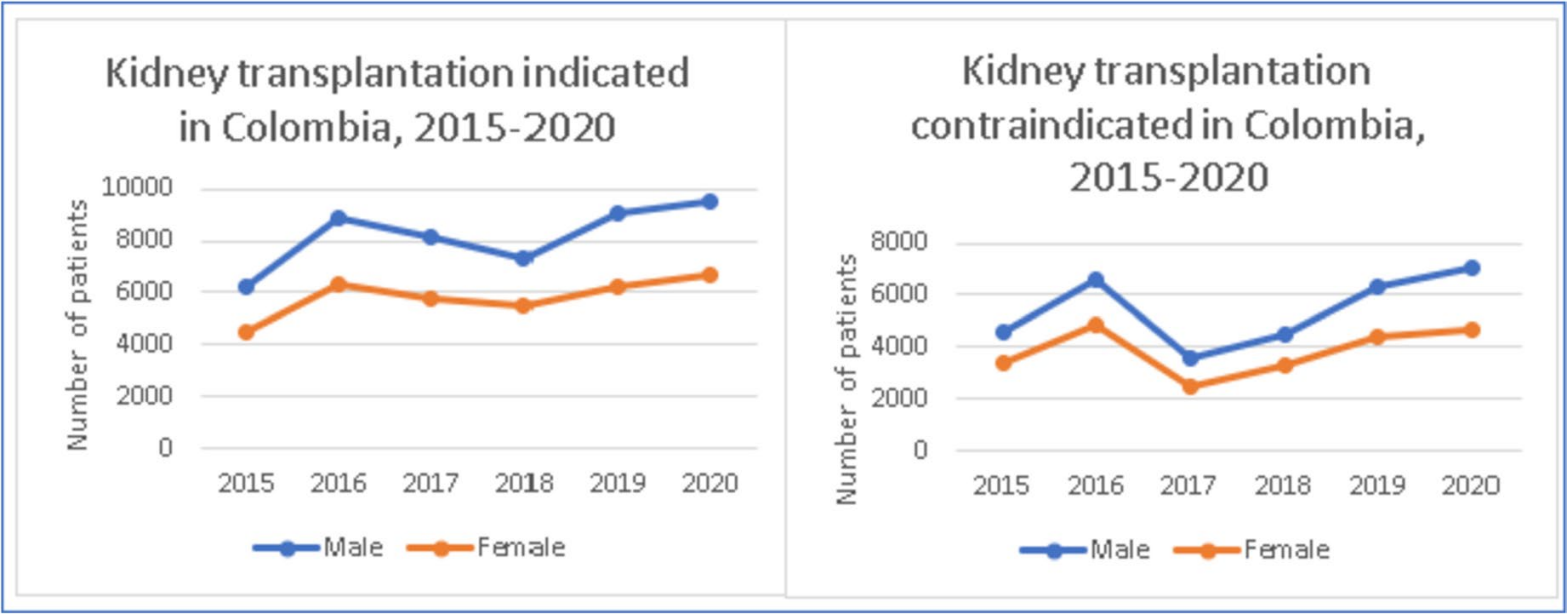
Patients indicated, and contraindicated for kidney transplantation in Colombia, 2015–2020

It is important to note that there is a marked difference between males and females when considering access to transplantation [5]. For example, in Colombia, 2741 patients are included in the kidney transplant waiting list, 42,8% of them are female patients (*n* = 1172). However, when considering referral for evaluation for kidney transplantation, there are more males (*n* = 9,502; 58,6%) than females (*n* = 6,270 patients; 41,4%) (Fig. 6). Also, 251 deaths were recorded in the waiting list, 38% females.

When analyzing gender differences in contraindication for KT in Colombia, unadjusted numbers show that there are more male patients being contraindicated than their counterparts.

### Living and cadaveric donors

#### Living donors

**Fig. 7:**
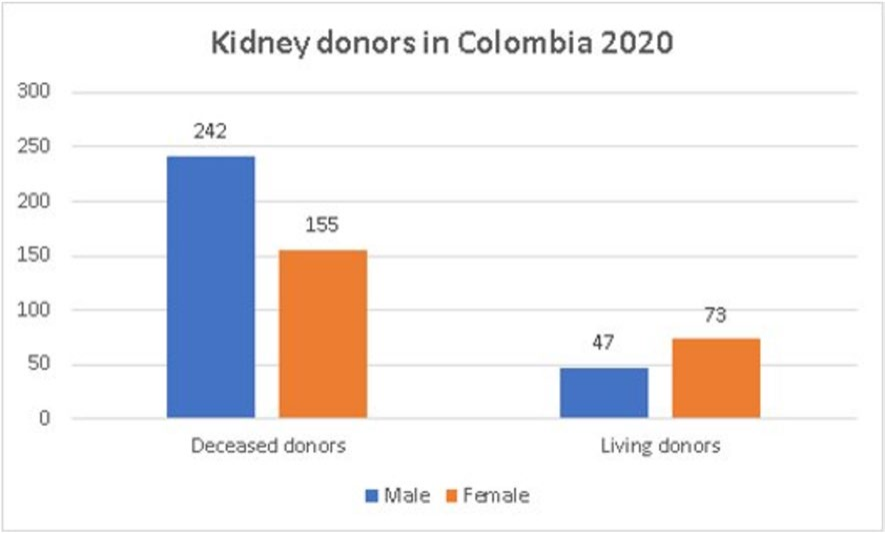
Kidney donors in Colombia 2020

In Colombia, over 2020 registered more female living donors (*n* = 73; 60,6%) when compared to male donors (*n* = 47; 39,4%) (Fig. 7).

#### Deceased donors

Consistent with what has been reported in the literature, deceased donors in Colombia are predominantly male (*n* = 242; 61%) vs. female (*n* = 155; 39%) (Fig. 7).

### Kidney transplantation

**Fig. 8:**
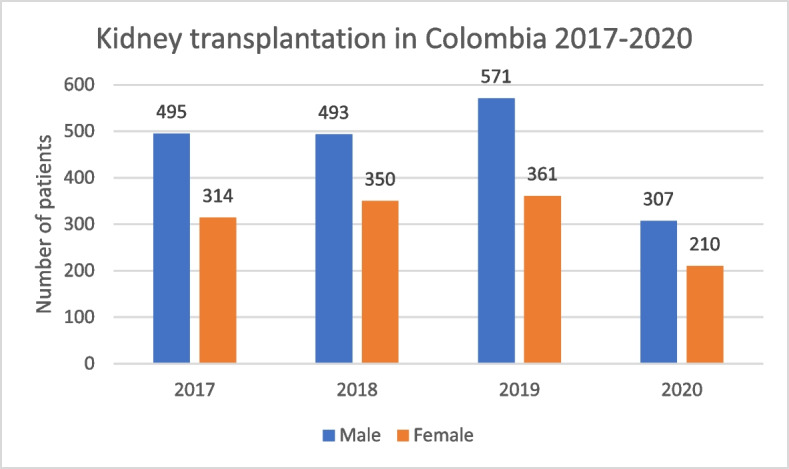
Kidney transplantation in Colombia 2017–2020

During the last four years (2017–2020) of available data, in Colombia, it has been reported a greater number of male patients receiving kidney transplants compared to female patients. For 2020, 307 male patients were transplanted, corresponding to 59,4% of the total (Fig. 8).

**Fig. 9:**
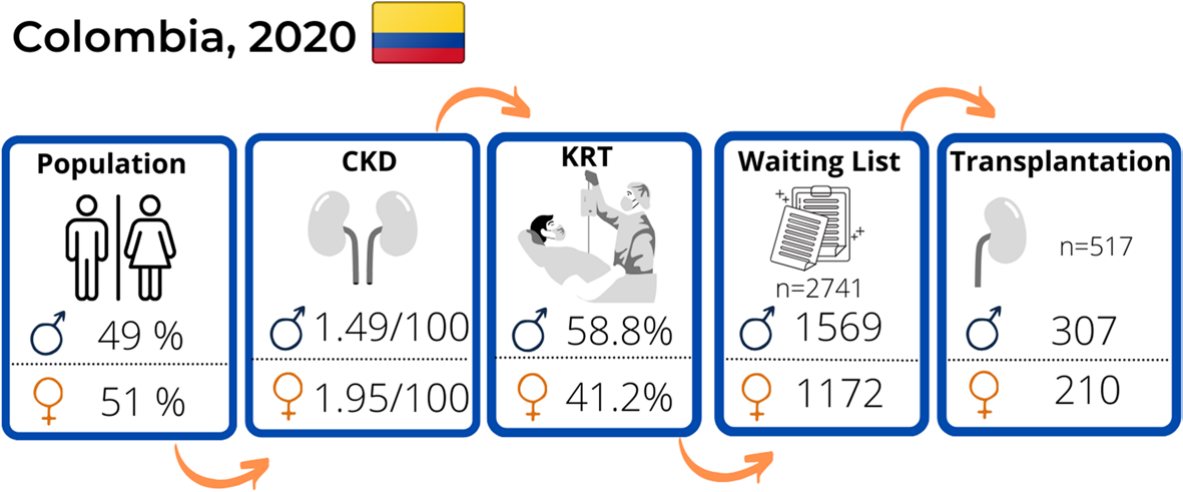
General characteristics by sex of the population in the CKD pathway

In Colombia there are more females in the general population and with ESKD, nevertheless there are less females in the waiting list and transplanted annually as shown in Fig. 9.

**Fig. 10:**
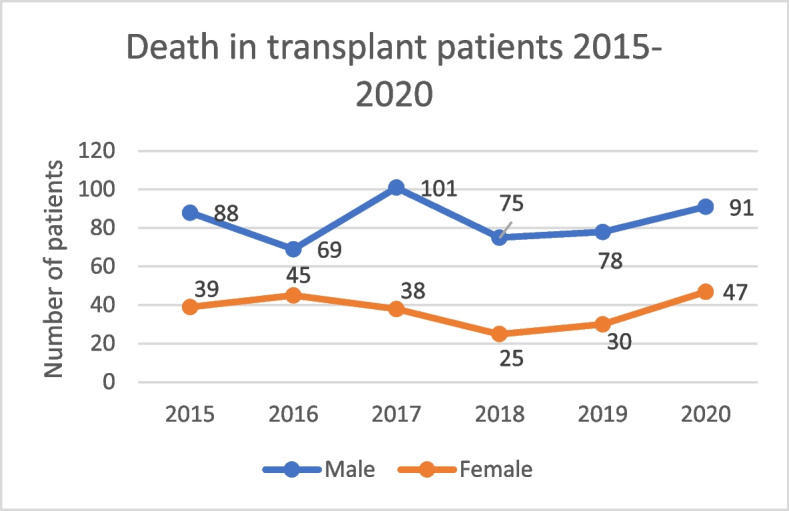
Mortality in KT patients in Colombia 2015–2020

Mortality in KT patients have consistently been higher in male patients when compared to female patients in Colombia (Fig. 10).

## Discussion

In Colombia, as observed in various other countries, a higher prevalence of ESKD has been documented in males. For example, in Italy, reports indicate a greater incidence of ESKD necessitating kidney transplantation in males compared to females. The hypothesized explanation for this disparity revolves around a higher occurrence of chronic hypertension and cardiovascular ischemic disease in the male population. It is posited that the proportion of females in stage 4 CKD exceeds that of males, a phenomenon attributed to the extended life expectancy of women and the potential overdiagnosis of CKD stemming from the utilization of formulas for estimating the glomerular filtration rate (GFR) [4]. Additionally, this trend may be linked to older females with CKD, who are more likely to choose conservative disease management strategies [4]. Notably, the specific details regarding this aspect are not accessible in our national database.

Even though the chronic kidney disease is more prevalent in females, women are less likely to be considered for KRT [4, 5]. Access to KRT has been shown to be inequitable between women and men in different parts of the world [6]. For our population, there are consistently more male populations in either kidney replacement therapies. Even though there are no gendered differences in the literature when analyzing the KRT modality, women have a higher probability of catheter use for dialysis initiation vs. arteriovenous fistula in males [5].

Women initiate dialysis with a lower GFR than men [5], although literature regarding the analysis of the *US Renal Data System (USRDS)* has reported a similar proportion of female and male patients receiving health care from a nephrologist. Although data is currently unavailable in Colombia, investigating this aspect further could yield valuable insights.

Inclusion in the waiting list concretes after evaluating each patient risk and the predicted survival benefit. Anyway, this evaluation can be subjective and complex, given the high prevalence of multiple comorbidities within the CKD population and the sparse evidence in this area [2]. Studies have shown that female patients have a lower probability of being included in waiting lists for transplantation [2, 12] and longer time before entering the waiting list [12]. This disparity is higher in older, unemployed, and diabetic women [12]. For older women with diabetes, the probability is 60% less when compared to males with diabetes and older age [2, 12].

Literature has shown that after adjusting for comorbidities, age and occupational status, females have 31% less probability of entering the waiting lists [12]. In Germany, there is a report of 18% [2]. These disparities endure even after accounting for confounding variables such as antibodies, comorbidities and CKD etiology [3].

Researchers in Mexico have found that the proportion of female patients included in waiting lists is only 40% [13]. In line with this observation, the average waiting time is shorter for males compared to females: 1.51 years (IQR 0.51–3.04) versus 1.61 years (IQR 0.62–3.31), with a statistically significant difference (*p* = 0.001) [13]. According to the *United Network of Organ Sharing (UNOS)*, 103,156 patients are included in the United States waiting list, and Euro transplant has 11,105 patients included, 61% of these patients are male [3]. In Colombia, as is consistent globally, female patients constitute only 44% of the waiting list. It is noteworthy that diverse reasons contribute to females avoiding inclusion on these lists, including fear, negative experiences witnessed in others, a preference to continue with dialysis, familial considerations, and cultural or religious factors [2].

In a 2023 study conducted in Argentina, their findings revealed a higher prevalence of chronic kidney disease (CKD) in women compared to men. However, this prevalence diminishes across the CKD stages, culminating in a notable increase in men reaching the end stage kidney disease (ESKD) requiring dialysis. Notably, access to transplant (ATT) is more prevalent among men than women, although post-transplant survival rates exhibit no discernible gender differences [14].

In Colombia, the prevalence of female living donors surpasses that of males, aligning with the trends reported in the existing literature. In a study made in Mexico, living donors are predominantly female (53.1% vs. 46.9%, *p* = 0.0001). This has been consistently reported in the literature around the world [3, 5, 6, 13, 15–19]. Official global information informs that 6 of every 10 living donors are female [6]. Nevertheless, 64% of the transplant recipients are male [5, 16]. When considering spouses as the source of living donation, only 6.5% of males donated, in contrast to 36% of wives who proceeded with the donation [16].

Similarly, mothers exhibit a higher likelihood of donating to their children, further contributing to the observed difference between men and women [16]. Upon examination, these mothers displayed a clear decision-making process, devoid of health-related concerns, and approached donation with a positive and optimistic attitude, refraining from characterizing it as a heroic deed [6].

In certain countries, there exists a perception that being a living donor is considered a “role for women.” This phenomenon has been identified and labeled as the domestication and feminization of organ donation in Mexico, as highlighted by Crowley-Matoka [13].

Deceased donors are predominantly male (64% vs. 36%, *p* = 0.0001) [3, 13]. Colombia is no exception. This can be a reflection of higher rates of traumatic death in young males [3]. Female donors are usually older and with cerebrovascular accidents as a cause of death [3].

In the domain of non-medical determinants affecting transplantation access, longstanding gender disparities favoring males have been consistently reported in academic literature, dating back several years and persisting consistently in the context of the United States [12, 20].

Nowadays, men are transplanted more when compared to women around the world. Kidney transplant recipients are predominantly male in Colombia (59.4%). This is consistent with reports in Mexico, in which 60% of the transplanted patients are male [13]. Even though the number of transplanted patients has increased in females, the difference between women and men has only intensified [13]. UNOS states that 60% of the transplanted patients are male and in Europe this percentage is even higher (62%) [3].

Studies indicate that women have a lower likelihood of receiving a transplant from a deceased donor [4, 6]. Female patients also have fewer conversations with healthcare personnel about KT when compared to male counterparts [6]. This holds significance, particularly since many women undergo pretransplant studies relatively late, rendering them ineligible for timely inclusion in waiting lists [6].

When studying transplant outcomes and survival models, adjusted to race, CKD etiology, dialysis duration pre-transplantation, donor age, body mass index, recipient weight, sensitization, when the donor is male and the recipient is female, regardless of age, has a higher risk of graft loss [3]. This difference accentuates in children and decreases in patients older than 45 years old. When the donor is female, only adolescent girls and young female adults (15–24 years old) had a higher incidence of graft loss when compared to boys and young male adults of similar ages. When the donor is female and the recipient is older than 45 years old, there is a lower risk of graft loss when compared to a male of the same age [3]. There exists a different hypothesis about the diverse aspects which could contribute to these observations, which include immunological reaction in female recipients to HY antigen (male tissues) [21], the immunostimulatory effect of estrogen [3, 4, 7] and the opposed immunosuppressive effect of testosterone [3, 4].

There is conflicting evidence as some studies have shown that male recipients have a worse prognosis when compared to females, which can be explained by a better adherence to treatment, assistance to follow-up appointments, lifestyle changes and a better attitude towards graft protection [3, 15].

### Factors which can influence gender disparity in organ transplantation

**Fig. 11:**
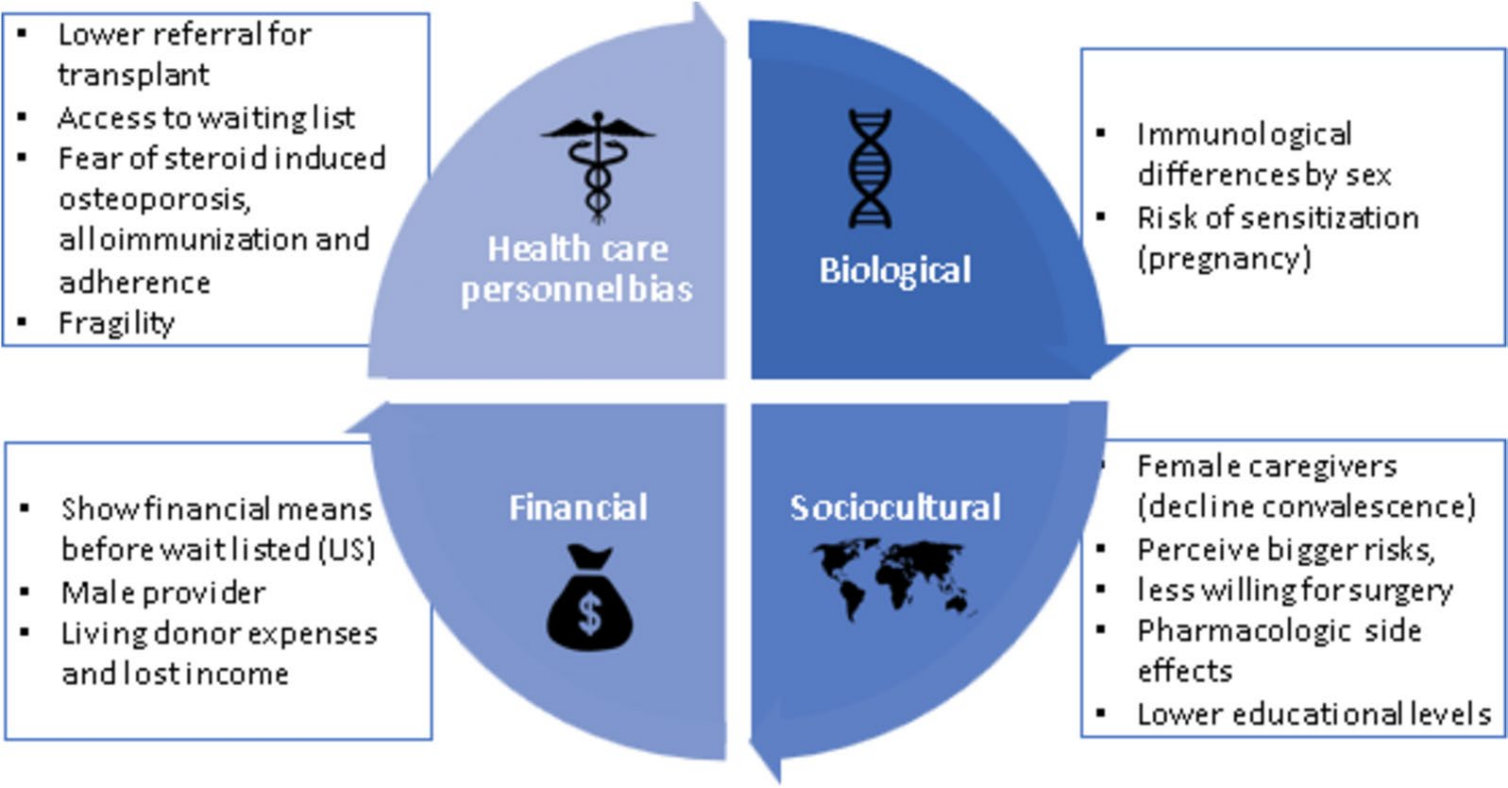
Identified barriers in KT

Various reports have sought to investigate the causes behind gender disparities in transplantation. These can be categorized into four groups: clinical, sociocultural [12, 17] financial and health worker’s bias (Fig. 11), nevertheless, these barriers have not been studied in the Colombian population.

a Biological
  Immunological differences by sex: women have shown to have a higher risk of sensitization after pregnancy [22], a barrier for transplantation.
  Lower prevalence of female in dialysis when compared to mail, regardless of the modality.
b Sociocultural
  Cultural role for females as a caregiver, which represents a motive to decline the convalescence after a transplantation.
  Females are regarded to have higher altruism and volunteerism than males, which is represented as a self-sacrificing nature.
  Women can perceive bigger risks and be less willing to be taken to surgery, as well as less keen of having immunosuppressive side effects [12]. Women give up aggressive treatment more.
  Lower educational levels may result in lower proportion of females accessing waiting lists in Europe [2]. This is because there is less knowledge about health, limited comprehension of the disease, diminished participation in decision making and restricted communication with health care workers [2].
  Lower social status may also contribute to gender disparities in this field.
  Patriarchism and low self-esteem in women, associated to indirect pressure of family or society to donate, may reflect as a stigma given by society for women to donate.
c Financial
  In nations such as the United States, prospective patients are required to demonstrate financial capacity as a prerequisite for placement on the waiting list [12]. Nevertheless, in countries such as Germany [1] and Colombia this is not an issue as we have universal health coverage.
  A higher probability that the male is the head of the family and therefore the provider (bread winner). If the female is transplanted, the male will have to lose some days work to take care of her. This fear for financial loss is critical.
  Women are more financially dependent on males.
  Living donor expenses, associated to lost income, may contribute to the low incidence of male living donors when compared to females [16].
  There are structural and/or administrative barriers for patients to get waitlisted and transplanted throughout the pathway of the kidney transplant journey.
d Health care personnel bias
  A bias within the health care personnel, which represents lower referral for transplant evaluations for women, the probability to be included on waiting lists and the lapse between the evaluation and the access to the waiting list [12].
  Bias in considering women for transplantation can be with reference to fear of corticoid induced osteoporosis, alloimmunization [5] and adherence [12].
  There is also a higher proportion of clinical health workers who can consider female patients as fragile [2].
  With less representation of women in healthcare personnel, there may be less empathy within the physician-patient dynamics and communication.

Even when a number of barriers have been shown to represent many different causes of gender disparity, there are no laws or policies regarding these [16]. Knowledge around gender disparity in transplantation is important to delineate the underlying mechanisms in CKD, access to treatment and outcomes. Though studies like this and others, we will be able to propose specific adaptations of sex and gender and innovate in day-to-day practice which could end in tailored health care for each patient.

It would also be important to verify the administrative process behind the kidney transplant patient journey, especially with the low numbers of waiting list despite the big numbers of indicated patients for transplantation. It is inevitable to have differences in proportions of values by gender, nevertheless it is our duty to search for different strategies to deconstruct the steps and diminish these differences if encountered. Additionally, it is important to evaluate if the social barriers described in the literature worldwide apply specifically to our population, as well as the economic role of the female as householder.

This may represent diverse barriers which should be identified and addressed accordingly to improve female access to kidney replacement therapy and posterior transplantation. As it has been done previously in Asia and the Pacific countries (ASTREG-WIT-KT) [23], the development of a Latin-American registry is fundamental to study in deeply this important theme, to achieve this, the involvement of international societies, namely Women in Transplantation (WIT), the Latin-American society of nephrology and hypertension (SLANH) and the Latin-American and the Caribbean society of transplantation (STALYC), is imperative. Using data to drive change is fundamental.

## Limitations

This is a retrospective, descriptive study based on the information of official government information, which constitutes a secondary bases analysis. We acknowledge this type of analysis carries out different measurement biases and does not identify causality, as it is not an analytic study. It is important to point out this official published data is the only annual information published regarding the whole Colombian population, and there are no other national data bases to triangulate information and verify its validity.

## Conclusion

In Colombia there are more females in the general population and with ESKD, nevertheless there are less females in the waiting list and transplanted annually. Therefore, women are less likely to be waitlisted or receive a KT. Nevertheless, there are important bias to be noted, there is a significant percentage of patients contraindicated for transplantation that could generate measurement biases and could be re-evaluated. Additionally, it would be interesting to use robust epidemiological methods to make an accurate diagnosis of the disparities. There are challenges throughout the process which contemplates transplantation as an endpoint.

### Supplementary Information

**Supplementary Material 1.**

## Data Availability

The raw database is available through the *Cuenta de Alto Costo* from Colombia as public data. The access link is as follows: https://cuentadealtocosto.org/.

## Abbreviations

CKD: Chronic kidney disease
KRT: Kidney replacement therapy
ESKD: End stage kidney disease
KT: Kidney transplant
eGFR: Estimated glomerular filtration rate
UNOS: United Network of Organ Sharing
ASTS: American Society of Transplant Surgeons
WIT: Women in Transplantation
SLANH: Latin- American society of nephrology and hypertension
STALYC: Latin-American and the Caribbean society of transplantation
